# Ancient human mitochondrial genomes from Bronze Age Bulgaria: new insights into the genetic history of Thracians

**DOI:** 10.1038/s41598-019-41945-0

**Published:** 2019-04-01

**Authors:** Alessandra Modi, Desislava Nesheva, Stefania Sarno, Stefania Vai, Sena Karachanak-Yankova, Donata Luiselli, Elena Pilli, Martina Lari, Chiara Vergata, Yordan Yordanov, Diana Dimitrova, Petar Kalcev, Rada Staneva, Olga Antonova, Savina Hadjidekova, Angel Galabov, Draga Toncheva, David Caramelli

**Affiliations:** 1 Department of Biology, University of Florence, Florence, Italy; 2 Department of Medical Genetics, Medical University of Sofia, Sofia, Bulgaria; 3 Department of Biological, Geological and Environmental Sciences, University of Bologna, Bologna, Italy; 4 Department of Cultural Heritage, University of Bologna, Ravenna, Italy; 5 Institute of Experimental Morphology, Pathology and Anthropology with Museum, Bulgarian Academy of Sciences, Sofia, Bulgaria; 6 The Regional Historical Museum of Sliven, Sliven, Bulgaria; 7 The Regional Historical Museum of Stara Zagora, Stara Zagora, Bulgaria; 8 The Stephan Angeloff Institute of Microbiology, Bulgarian Academy of Sciences, Sofia, Bulgaria

## Abstract

One of the best documented Indo-European civilizations that inhabited Bulgaria is the Thracians, who lasted for more than five millennia and whose origin and relationships with other past and present-day populations are debated among researchers. Here we report 25 new complete mitochondrial genomes of ancient individuals coming from three necropolises located in different regions of Bulgaria – Shekerdja mogila, Gabrova mogila and Bereketska mogila – dated to II-III millennium BC. The identified mtDNA haplogroup composition reflects the mitochondrial variability of Western Eurasia. In particular, within the ancient Eurasian genetic landscape, Thracians locate in an intermediate position between Early Neolithic farmers and Late Neolithic-Bronze Age steppe pastoralists, supporting the scenario that the Balkan region has been a link between Eastern Europe and the Mediterranean since the prehistoric time. Spatial Principal Component Analysis (sPCA) performed on Thracian and modern mtDNA sequences, confirms the pattern highlighted on ancient populations, overall indicating that the maternal gene pool of Thracians reflects their central geographical position at the gateway of Europe.

## Introduction

Bulgaria is situated in the eastern part of the Balkan Peninsula, at the connection point between Southeastern Europe, Eurasian steppe, Anatolia and the Aegean islands. The presence of modern humans in this region is attested starting from 40 kya by the Paleolithic series at Bacho Kiro and Temnata Dupka Caves^1–3^. Some archaeological sites associated with early farmers, as well as the earliest evidence of copper metallurgy in Europe, indicates that this area played a significant role both in the Neolithic and in the Metal Ages^4^. One of the best documented Indo-European civilizations that inhabited Bulgaria consists in the Thracians, whose cultural legacy is still evident in the modern society.

Different theories have been historically proposed about the origin of the Thracians. Today it is assumed that the Thracian culture emerged and formed in the early Bronze Age^5–7^, a period characterized by strong cultural changes and movements of people westward from the Steppe^8^. During the 5th and 4th millennium BCE, the inhabitants of the eastern region of Balkans were organized in different groups of indigenous people that, over time, were named under the single ethnonym of “Thracians”^9–11^. According to historical and archaeological sources, the Thracian culture flourished during the 2nd and 3rd millennia BCE^12,13^. The rich cultural and historical heritage, represented by fortresses and necropolises, as well as by the world-famous Panagyurishte, Valchitran, Lukovit and Rogozen treasures, dates back to this period. In the later periods, several populations (Greeks, Macedonians, Slavs and proto-Bulgarians) arrived in the Balkans, reaching the lands occupied by Thracians and mixing with them, thus influencing their cultural and biological identity^11^.

Genetic analyses on both autosomal variations^14^ and uniparental genetic markers^15–17^ of present-day Bulgarians, locate them between Eastern European and Mediterranean populations, with a particular affinity to the neighboring groups from Greece and the Balkans^16^. In addition, the Bulgarian maternal genetic pool particularly suggests a major Western Eurasian origin, tracing their ancestry to lineages that witness a complex genetic structure of the region today and reflect different peopling and admixture events from the Upper Paleolithic to the onset of the Neolithic and Post-Neolithic in Europe^15^. Recent genome-wide ancient DNA (aDNA) based studies on Southeastern Europe, have shown that Neolithic population from present-day Bulgaria was closely related with the northwestern-Anatolian-Neolithic ancestry that signals the spread of Early farmers across Europe, except for the individuals lived in the mid-sixth millennium BC in Malak Preslavets, who revealed a significantly higher level of hunter-gatherer-related ancestry than the other Balkan Neolithic individuals^18^. Starting from the early 3rd millennium BCE, migrations from the adjacent Pontic-Caspian and Eurasian steppe also played an important role in the transformation of the European genetic landscape, and the contribution of Steppe ancestry to Southeastern European populations increased particularly during the Bronze Age^18^. Although our understanding of the population and cultural dynamics occurred in the (pre-) history of Balkan Peninsula is starting to be increasingly elucidated, the genetic details on the local civilization remain unknown and this information is only partly available for the ancient (proto-) Bulgarian people^18,19^. We now have the opportunity to investigate the genetic structure of the Thracians, an ancient people that lasted for more than five millennia and whose origin and relationships with other past and present-day populations are still debated among researchers.

**Figure 1:**
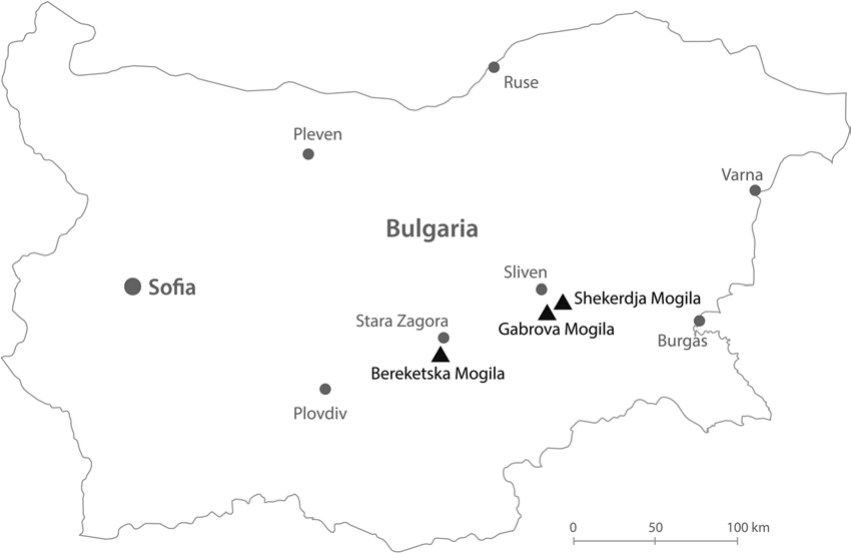
Geographical location of the necropolises in Bulgaria. Black triangles specifically indicate the locations of the considered archaeological sites while the grey dots refer to current Bulgarian provinces. The map is plotted using https://www.freepik.com/, processed with Adobe Illustrator CS6 and modified with Photoshop CS6 (2012) by Dimitar Spassov - web developer (dimitarspassov@gmail.com) and Desislava Nesheva. Image is attributed to valeria_aksakova/Freepik.

To investigate the genetic structure and population history of this ancient civilization, we analyzed 25 complete mitochondrial genomes from three Thracian necropolises (Fig. 1 and Table 1) along with modern and ancient European data. The characterization of the Thracian mitochondrial DNA (mtDNA) variability may have important implications for understanding the dynamics of interaction between Eastern Europe and the Mediterranean, and will also contribute to better clarify the genetic evolution of European populations and the origin of contemporary Bulgarian gene pool.

**Table 1:** Sample analyzed.

Sample ID	Grave	Element	Dating/Chronology
**Shekerdja mogila**			
*SM 4*	4	tooth	Early Bronze age
*SM 8.1*	8	long bone	2462-2197 calBCE (3839 ± 45 BP, LTL16867A)
*SM 10.1*	10	tooth	Early Bronze age
*SM 24.1*	24	tooth	Early Bronze age
*SM 24.2*	24	tooth	Early Bronze age
*SM 24.3*	24	long bone	Early Bronze age
*SM 24.4*	24	long bone	Early Bronze age
*SM 25.2*	25	long bone	Early Bronze age
*SM 31.2*	31	long bone	Early Bronze age
***Gabrova mogila***			
*GM 9*	9	long bone	Early Bronze age
*GM 23*	23	long bone	Early Bronze age
*GM 28*	28	long bone	Early Bronze age
*GM 30.2*	30	long bone	Early Bronze age
*GM 30.3*	30	long bone	3348-3010 calBCE (4463 ± 45 BP, LTL16866A)
*GM 30.4*	30	long bone	Early Bronze age
***Bereketska mogila***			
*BM AG*	AG	tooth	Early Bronze age
*BM 2*	2	tooth	Early Bronze age
*BM 3*	3	tooth	Early Bronze age
*BM 5*	5	tooth	Early Bronze age
*BM 6*	6	tooth	Early Bronze age
*BM 9*	9	tooth	Early Bronze age
*BM 10*	10	tooth	Early Bronze age
*BM 13*	13	tooth	Early Bronze age
*BM 15*	15	tooth	Early Bronze age
*BM 24*	24	tooth	Early Bronze age
*BM 31*	31	tooth	Early Bronze age
*BM 36*	36	tooth	Early Bronze age
*BM 40*	40	tooth	Early Bronze age
*BM 44*	44	tooth	2197-2166 calBCE (3671 ± 45 BP, LTL16870A)
*BM 46*	46	tooth	Early Bronze age
*BM 51*	51	tooth	Early Bronze age
*BM 51A*	51	tooth	Early Bronze age
*BM 58A*	58	tooth	Early Bronze age
*BM 59A*	59	tooth	Early Bronze age
*BM 61*	61	tooth	Early Bronze age
*BM 68*	68	tooth	Early Bronze age
*BM 69*	69	tooth	Early Bronze age
*BM 73*	73	tooth	Early Bronze age
*BM 76*	76	tooth	Early Bronze age

For each sample number of grave, anatomical element and chronology. For samples SM 8.1, GM 30.3 and BM 44 radiocarbon date are reported.

## Results

We successfully reconstructed complete or almost entire mitochondrial genomes for 26 individuals, 3 from Shekerdja mogila, 1 from Gabrova mogila and 22 from Bereketska mogila (Table 2). All the resulted sequences reach the standard quality requested to guaranty the reliability of the NGS data; CtoT patterns range between 20% to 46%, average fragment size vary from 44.4 base pair (bp) to 67.4 bp and no significant levels of present-day human contamination were detected (Table 2). Only one sample, BM-51, showed a high level of contamination and was not considered in the following statistical analyses.

**Table 2:** Sequencing and mapping summary.

Sample ID	Raw reads	Merged reads (%)	Mapped reads prior rmdup	Mapped reads after rmdup	Contamination estimate		mtDNA Average Coverage Depth	mtDNA Average Coverage (%)	Deamination pattern		Average fragment length (bp)	Hg
					First iteration (%) [low-high]	Final iteration (%) [low-high]			5′ (%)	3′ (%)		
*SM 4*	1042513	975974 (93.62)	14659	10820	0 [0–0.05]	2 [1–3]	35.73	98.36	41.19	40.68	54.7	J1c
*SM 8.1*	291072	280395 (93.33)	17037	11026	0 [0–1.5]	1 [0–2]	31.01	98.05	46.00	49.32	46.6	U5a1a2b
*SM 24.2*	138644	133841 (96.54)	8598	7666	0 [0–0.5]	1 [0–2]	20.92	97.63	35.90	35.90	45.2	HV1a’b’c
*GM 30.3*	140659	133681 (95.04)	4692	4194	5 [2–8]	2 [1–3]	13.61	95.38	42.46	41.33	53.8	K1c1
*BM AG*	252894	242542 (95.91)	67931	52866	0 [0–0.5]	1 [0–2]	178.75	100	21.10	21.00	56.0	U5b2a1a1
*BM 2*	1129167	1072135 (94.95)	568403	362803	4.5 [4–5]	2 [1–3]	1299.92	100	20.00	20.00	59.4	N1b1a1
*BM 3*	455909	398419 (87.39)	125888	76387	0 [0–1]	2 [1–3]	310.93	100	23.33	22.51	67.4	H3ak
*BM 5*	241044	223147 (92.58)	44418	39517	6.5 [5.5–7.5]	1 [0–2]	155.55	100	25.95	24.28	65.2	H5a1a
*BM 6*	795728	767050 (96.40)	272955	170311	0 [0–1]	1 [0–2]	557.29	100	32.25	30.10	54.2	H7a1a
*BM 9*	306514	293319 (95.70)	38873	27053	0 [0–0.05]	1 [0–2]	102.67	99.96	32.74	31.10	62.6	H7
*BM 10*	1051289	1015983 (96.64)	190927	120090	8 [9–7]	1 [0–2]	411.12	100	24.67	23.51	56.7	U4c2a
*BM 15*	308266	292881 (95.01)	67093	43783	0 [0–0.05]	1 [0–2]	170.28	100	28.55	26.10	64.4	T2b
*BM 24*	336874	306128 (90.87)	48355	39312	5.5 [4.5–6.4]	1 [0–2]	136.90	99.98	31.07	27.00	57.5	I2
*BM 31*	291132	259451 (89.12)	35740	31783	57 [56–58]	16 [15–17]	122.07	97.04	29.24	26.46	63.6	J1c9
*BM 36*	542629	521894 (96.18)	244787	178811	0 [0–0.1]	1 [0–2]	599.48	100	33.24	31.47	55.6	N1
*BM 40*	199895	190493 (95.30)	45850	40103	0 [0–0.05]	2 [1–3]	130.67	99.97	28.72	29.12	54.0	T2e2a
*BM 44*	167444	162418 (97.00)	40293	32973	0 [0–0.05]	2 [1–3]	100.58	99.93	34.99	33.01	50.5	HV0
*BM 51A*	1025635	971945 (94.77)	27811	289429	46.5 [45.5–47.5]	10 [9–11]	84.19	99.06	35.98	31.63	56.9	K1c1
*BM 58A*	164453	156982 (95.46)	39604	33144	0 [0–0.05]	2 [1–3]	113.77	99.97	30.82	28.72	56.9	K1c1
*BM 59A*	319622	303936 (95.09)	95874	77103	0 [0–0.05]	2 [1–3]	258.96	100	30.16	29.86	55.6	T2b
*BM 61*	240732	230927 (95.93)	63892	56475	7 [6–8]	1 [0–2]	186.98	99.97	29.29	28.74	54.9	J1c6
*BM 68*	518653	503547 (97.09)	174125	126713	5 [2–8]	3 [4–2]	400.39	99.98	33.31	32.98	52.4	K1c1
*BM 69*	361277	343403 (95.05)	70252	55118	0 [0–0.05]	1 [0–2]	165.64	100	32.95	30.15	49.8	H5b
*BM 73*	311752	292048 (93.68)	127325	95877	0 [0–0.05]	1 [0–2]	256.74	100	37.23	35.02	44.4	H76a
*BM 76*	486546	473556 (97.33)	86904	61483	0 [0–0.05]	1 [0–2]	192.58	100	22.55	23.35	51.9	H4a1

Number of raw reads, number of merged reads, number of mapping reads before and after removing PCR duplicates, contamination estimate (Schmutzi), average depth of coverage, mitochondrial coverage, deamination pattern (Schmutzi), average fragment length and mt haplogroup (assigned with HaploGrep) are reported.

**Figure 2:**
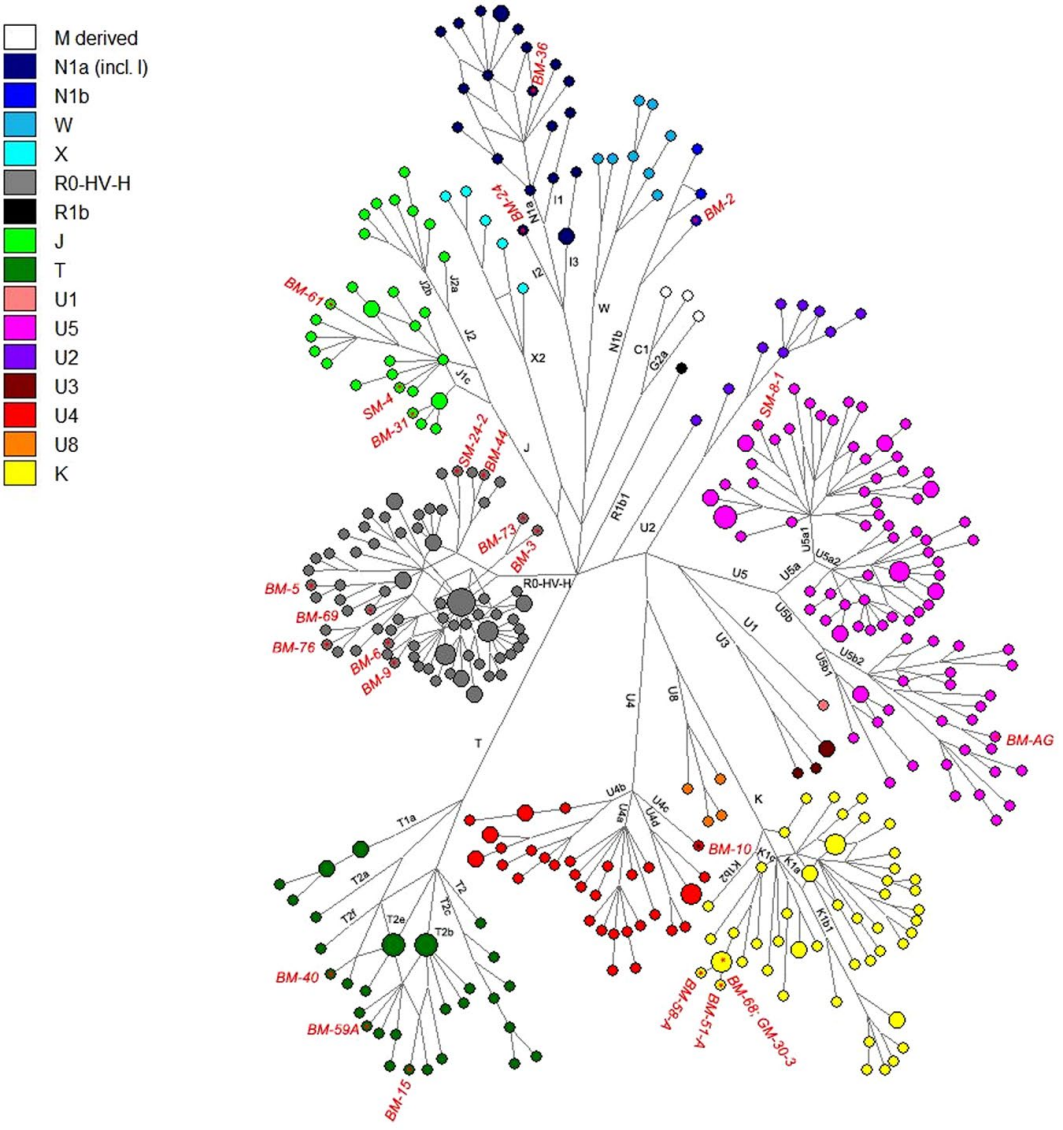
Median Joining Network representing the phylogenetic relationships between the new Thracian samples (highlighted in red labels) and the ancient reference dataset. Major mitochondrial lineages and sub-lineages are indicated by different colors as specified in the legend at the top-left.

The direct radiocarbon dating performed on the samples BM 44, SM 8.1 and GM 30.3 placed the remains at II-III millennium BC (CEDAD, Centro di DAtazione e Diagnostica, Univerità del Salento, Italy) (Table 1), that corresponds to the age estimated according to the archaeological record. The mtDNA sequences obtained were assigned to 21 different haplogroups, representative of the mitochondrial variability of Western Eurasia (Table 2 and Supplementary Table S1). Phylogenetic links between haplotypes of the Thracian samples and comparison ancient data are shown in the Median Joining Network (Fig. 2). Most of the Thracian individuals belong to sub-lineages of the macro-haplogroup H, which accounts for an overall frequency of 33%. This is the most frequent mitochondrial lineage in present-day Europe, representing over 40% of the total mtDNA variability^20^. Its frequency observed in the Thracians samples is almost similar to the frequency in contemporary European population. Two individuals belong to haplogroup HV, an ancient European lineage likely originating in the Mediterranean region during the Last Glacial Maximum (LGM)^21^. In ancient samples, HV has been identified in one Mesolithic specimen from Sicily^22^ and in early Neolithic remains from Spain^23^, Germany^8^ and Russia^18,24^; Mathieson *et al*.^18^ reported a HV haplotype in one sample from Serbia dating from 5800 BCE. Moreover, haplogroup HV was observed in Copper Age specimens from Scotland, Hungary and Germany^25^ and in Hungarian and Israeli samples from the Chalcolithic period^26,27^

We found four individuals belonging to haplogroup K1c (GM-30.3, BM-51A, BM-58A and BM-68). All the haplotypes contain the expected K1c defining variants with the following private polymorphisms: GM-30.3, 309.1T, 310C, 7441T and 16519C; BM-51A, 16519C; BM-58A, 310C, 513.1CA and 16519C; BM-68, 5297T and 16519C. Nowadays the highest observed European frequency of the lineage K is in Bulgaria (13.3%)^28^ and K1c is particularly common in Slavic-speaking countries. In ancient populations, the haplogroup K1c has been identified in six hunter-gatherers dated before the arrival of farming (one in Romania, three in Serbia^18^ and two in Greece^29^), in two Bronze-Age individuals from Hungary and Bulgaria^18,30,31^ and in two Central-Europe farmers associated with the Bell-Beaker culture^25,32,33^. The phylogenetic network analysis (Fig. 2) reveals that the detected K1c haplotypes in Thracians are closely related to hunter-gatherers from Iron Gates and Bronze Age individuals from Bulgaria and Hungary.

Three samples belong to haplogroup J1c (SM-4, BM-31 and BM-61). The SM-4 individual shows three personal transitions previously identified at positions 199C, 8730G and 13928A, and a private mutation at 13686G. The haplotypes of samples BM-31 and BM-61 fall within the sub-haplogropus J1c9 and J1c6, respectively. Currently, J1c, which dates to ∼16 ka ago, is found mainly in Europe, especially in Central Europe, Balkans and Ukraine, where it encompasses almost 80% of total J1 lineages. Pala *et al*.^34^ suggested that during the LGM, haplogroup J sub-lineages arose in the Near Eastern refugia and recolonized Europe following the end of the last glaciation. In particular, J1c is not yet found in any hunter-gatherers, and the oldest individuals belonging to this lineage were found in Iran^35^ and in Anatolia^30^ dating to 8000-7700 BCE. It is possible that J1c arrived in Thracia from Anatolia during the early stages of the Neolithic expansion. The expansion of farmers played an important role also in the diffusion of haplogroup T, which has been found in three Thracian samples with the T2b (BM-15 and BM-59A) and T2e (BM-40) sub-lineages. Pala *et al*.^34^ particularly suggested that these lineages entered Europe from Anatolia in the Late Glacial period, and have been later diffused around Europe by Neolithic agriculturalists after intermingling with the inhabitants of Southeast Europe. Overall, while haplogroups H, K, J and T arose throughout the Neolithic increasing frequencies in different later communities and present-day European populations, the haplogroup U sub-lineages including U2, U4, U5 and U8 instead mark the genetic pool of European pre-LGM hunter-gatherers^36–38^.

**Figure 3:**
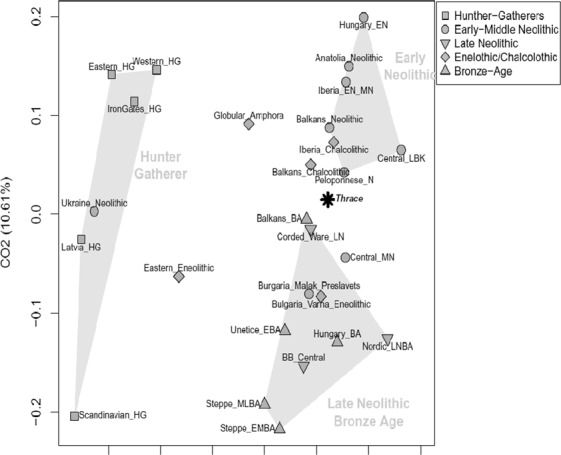
Correspondence Analysis (COA) based on mtDNA genomes from ancient populations. The available ancient mitogenomes were classified into geographically and culturally distinct groups as detailed in Supplementary Table S2. Ancient population groups from different periods were plotted using distinct symbols as specified in the legend at the top-right of the plot. The polygonal areas are intended at emphasizing the main clustering patterns emerged along the first (Hunter-gatherers) and second (Early Neolithic vs. Late Neolithic/Bronze Age) components as discussed in the Result section.

The mtDNA genetic relationships between Thracians and the other ancient Eurasian populations (Supplementary Table S2) were directly explored through a correspondence analysis (COA, Fig. 3). The first component, which accounted for 28.3% of the total variance, clearly separates all hunter-gatherers from the rest of Neolithic, Bronze Age and Iron-Age population groups. Along the second component (10,6% of variance), the ancient populations appear instead distributed along a cline of genetic variation which extends from the Early Neolithic farmers of Southern Europe and Anatolia to the Late Neolithic/Bronze Age Europeans and Steppe pastoralists, in accordance with the genomic structure of ancient Europe^29,30,32,33^. From an autosomal genetic perspective, besides showing the clear discontinuity of Paleolithic hunter-gatherers, recent genome-wide aDNA studies, have indeed outlined two opposite genetic components contributing to the European genetic ancestry: i.e. the ancestry of the Early European farmers related to Anatolian farmers and pre-farming Levant populations and, on the other side, the so-called Steppe ancestry eventually spread into Europe and Asia during the Bronze Age migrations of Yamnaya herders. In this scenario, the mtDNA genetic composition of analyzed Thracian population located them in the middle of this cline, clustering closely to the Peloponnese-Neolithic individuals (Peloponnese_N) and the Chalcolithic and Bronze Age populations of the Balkans (Balkans_Chalcolithic, Balkans_BA). This finding seems to support a mitochondrial genetic profile of the Thracians that reflects their geographical position at the gateway of Europe. In a more general perspective, Thracians show a mtDNA genetic composition that is thus intermediate between the western Eurasian and the Mediterranean populations, documenting a prolonged interaction between people of these regions during the Bronze Age. On the other hand, the relatively higher distance with the Bronze Age populations from the Steppe (Steppe_EMBA and Steppe_MLBA), may support the hypothesis that the Thracians largely derived from local people^9–11^ with only a low percentage of the gene flow from the Steppe, at least during the early stages of their cultural development. However, in order to better explore this hypothesis, it is worth emphasizing that the perspective offered here by the analysis of mitochondrial genomes should be integrated by the possibility of testing the results obtained with Y-chromosome and autosomal genome-wide data. At this respect, several studies have indeed pointed out the sex-biased nature of the recent demographic changes and expansions in Eurasia^39–43^, thus suggesting possible sex-specific patterns of migration.

**Figure 4:**
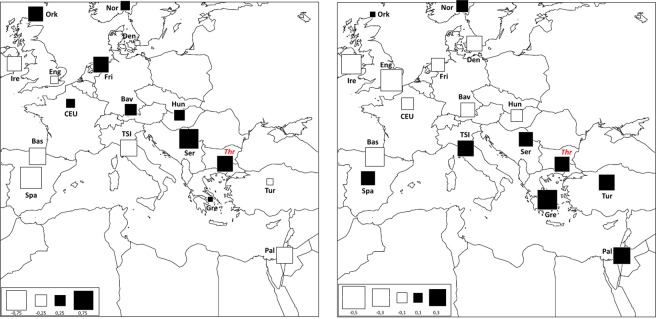
Spatial Principal Component Analysis (sPCA) based on Thracian and modern comparison populations. The first two global components sPC1 (**a**) and sPC2 (**b**) are depicted. Positive values are represented by black squares; negative values are represented by white squares; the size of the square is proportional to the absolute value of sPC scores.

In addition to a temporal frame, in order to explore the spatial pattern of mtDNA genetic variability, the genetic composition of past Thracian population was compared also with that of present-day human groups by means of a spatial Principal Component Analysis (sPCA, Fig. 4). Along the first component (sPC1) the ancient Thracians are closely related with Central-East European populations, while along the second component (sPC2) our samples show higher resemblance with present-day Mediterranean groups. Despite the general lack of statistical support to a clear-cut genetic structure (Gtest: obs  =  0.196, P-value  =  0.182), as expected due to the well-known higher genetic homogeneity of the mtDNA variability, this pattern reflects the one highlighted by COA analysis on ancient populations. Overall, the mitochondrial genetic structure observed in our sample seems to be mainly a consequence of demographic processes between two macro-areas: West Eurasia and the Mediterranean. This is in agreement with previous studies on modern samples^14–16^ that identify features of both Eastern Europe and Mediterranean area in Bulgarian population.

## Discussion

In the present study, we reconstructed and analyzed complete mitochondrial genomes from 25 Bronze Age individuals sampled in three Bulgarian necropolises. According to the archaeological records, these cemeteries are associated to the Thracians culture and the chronology, attributed by funerary context, was confirmed by three direct radiocarbon dating placing the remains at II-III millennium BC. These data were used to explore, for the first time, the genetic structure of this ancient population.

We found that the Thracian maternal gene pool is represented essentially by Western Eurasian haplogroups, as expected given the well-known overall mtDNA genetic similarity among all European populations. However, when we compared the complete mitochondrial sequences of Thracians to that of ancient and contemporary Eurasian populations, we observe that their genetic profile reflects their nexus geographical position between east and west.

Several studies demonstrated that Balkan Peninsula has been in different times a crossroad for people moving from and to Europe and beyond^16,44^. While previous analyses of modern populations demonstrated the impact of such migrations on the genetic makeup of present-day Bulgarians^14–16^, scarce information were available for the ancient (proto-) Bulgarian maternal gene pool and were mainly limited to HVS1 data from the medieval period^19^. In this study, we provide, for the first time, genetic details of an ancient population, which is particularly relevant from both a chronological and a geographical point of view. In accordance with their geographical location, Thracians show a genetic composition clearly intermediate between East Europe and Mediterranean, that suggests multiple admixture events and population movements occurred across what is now the modern day Bulgaria. Albeit limited to DNA transmitted along the female lines of descent, our genetic data on ancient Thracians provide a direct evidence of how the Balkan region has been a link between East and West Europe since the prehistoric time, and particularly during the Neolithic and post-Neolithic events. In this perspective, future studies will certainly benefit from the analysis of nuclear genome (Y-chromosome and autosomal genetic variation) in order to integrate the observed mtDNA genetic patterns within a more comprehensive overview and for testing the possibility of different sex-biased migrations in the area.

Overall, the ancient mtDNA data presented in this study integrate the existing database and has important implication for understanding the origins of the peopling in this part of Europe and for enlarging the knowledge on the ancient Bronze Age civilizations. How and to what extent ancient Thracian people has contributed to the present-day Bulgarian gene pool remain largely unknown due to the lack of large mitogenomes from contemporary populations from the area, necessary for a phylogenetically and demographically informative comparison.

## Methods

### Archaeological background and sample information

We processed 41 archaeological human remains, retrieved from three necropolises located in different regions of Bulgaria: Shekerdja mogila (SM), Gabrova mogila (GM), and Bereketska mogila (BM) (Fig. 1, Table 1). According to the archaeological features, funerary rites, grave goods and directed radiocarbon dates, the investigated individuals are all attributed to the Thracian culture.

The tumulus Shekerdja mogila is located near to the village of Kamen, 1 km north of the Sliven region in east-central Bulgaria. This necropolis is a mass grave in which many Early Bronze Age remains have been discovered. Funerary objects and a body in the hocker position (fetal-like position where the arms embrace the lower limbs), characteristic of the Thracian culture, were found in the southern side of the tumulus^45–47^. Grave No. 8 (examined sample SM 8) is a rectangular pit embedded in the mound embankment with a size of 0.48 per 1 m and a 0.21 m depth. The buried individual is a 3–4 years old child with remnants of red ochre on the bones and skull. Grave 24 (examined sample SM 24.2) is a mass grave; anatomically scattered bones and skulls of 7 individuals were found. One of the skulls has been placed in a large ceramic vessel. The anthropological research showed that the skeletons belong to two women (20–30 years old), one male (30–35 years old), three children (2.5 and 6 years old) and a neonate. Sample SM 24.2 belongs to 6 years old child with remnants of red ochre on the bones. Amulets of wolf/dog, short obsidian, clay pot and a fragmented ceramic bowl placed inside an urn were found close to the skeletons.

The tumulus Gabrova mogila is located near the Shekerdja mogila, north of the village of Kamen, Sliven region, east-central Bulgaria. This tumulus has many funerary and ritual objects typical of the Thracian period^45–47^. We analyzed 6 graves from the early Bronze Age. Grave No. 30 (examined sample GM 30.2) is located almost in the geometric center of the mound at 3.10 m from the central benchmark. The tomb pit has a rectangular shape and has east-west orientation. It is filled with dense loam soil and four adults were found at the bottom of the pit. The corpses are placed in a stretched position on their backs and their arms are bent in their elbows with slightly spaced legs. The buried individuals are male and the skeleton No. 2 (GM 30.2) is 20 years old. The inventory found in the grave consists of metallic, ceramic, bone and flint objects. A bronze ax was discovered, which is typical for the early Bronze Age. Amulets of wild boar teeth have been placed next to the skulls of the four skeletons.

The tumulus Bereketska mogila is the largest prehistoric necropolis in Bulgaria. It is located on the right bank of Bereketska River, in central Bulgaria. So far, this tumulus is the only flat necropolis from the Early Bronze Age that has been studied^48,49^. The burials excavated showed individuals in hocker position on the right and left side, in a bent position of the back, in double and multiple inhumations. Funerary context, as well as the presence of ochre and stone pounders undoubtedly bears witness to the contacts between Early Bronze Age Thracians and the North Pontic area, particularly the Yamnaya culture.

### Molecular analysis, NGS data processing and authentication

Molecular analyses of the anthropological samples were performed in the Laboratory of Molecular Anthropology and Paleogenetics, University of Florence, following strict guidelines and standard precautionary measures to avoid contamination during all experiments. The sampling of bone powder was conducted using a microdrill, selecting the compact bone from the inner part of long bones and the dentine part from teeth. Fifty milligrams of bone or dentine powder were used for DNA extraction using a silica-based technique that allows ancient DNA molecules to be efficiently recovered^50^. DNA libraries were prepared from the extracts following a custom double-indexing protocol^51,52^ optimized for ancient samples, in order to make the DNA immortalized, barcoded and available for the Next Generation Sequencing (NGS). Negative controls were processed during each experimental step. A target enrichment strategy was followed to select the DNA molecules attributed to the mitochondrial genome^53^. Enriched libraries were pooled in equimolar amount and paired-end sequenced (2 × 75 + 8 + 8 cycles) on Illumina MiSeq platform.

Sequences were demultiplexed and sorted according to the sample, and then raw reads were processed with EAGER^54^. Adaptor sequences were trimmed and paired-end reads were merged into single sequences with a minimum overlap of 10 bp, in order to exclude all the sequences derived from molecules longer than 140 bp. Only reads with a minimum length of 30 bp were kept. Filtered reads were mapped to the revised Cambridge Reference Sequence (rCRS, NC_012920.1) using CircularMapper, a mapping method especially designed for circular reference genomes; reads with mapping quality below 30 were discarded. PCR duplicates were removed using DeDup and consensus sequences for the mitochondrial genomes of all samples were called using schmutzi (parameters: “–logindel 1 –uselength”)^55^. DNA damage patterns at the ends of the molecules and average fragment length were taken into account to identify and call endogenous bases. Present-day human contamination estimates were performed using a non-redundant database of 197 human mitochondrial genomes available in the software package. Misincorporation patterns at the 5′ and 3′ ends were computed using contDeam, a program provided with the schmutzi package. A summary of the results for each analyzed sample is provided in Table 2. Mitochondrial haplogroups for each sample were determined using HaploGrep^56^ based on PhyloTree build 17^57^, followed by manual verification of each diagnostic variant (Supplementary Table S1).

Consensus sequences for each individual were submitted to NCBI GenBank under the Accession Numbers MH605025-MH605049.

### Population genetics analyses

To set the observed mtDNA variation into a wider genetic landscape and with the aim of investigating possible genetic relationships with both modern and ancient populations, the Thracian mitogenomes were compared with those of reference datasets extracted from the literature. The modern comparison dataset consisted of 320 individuals from 16 West Eurasian populations for which comparable mtDNA whole genome sequencing data were available^42^. In particular, we selected data from population-based mtDNA sequencing studies that allowed to maximize the representativeness of the European genetic landscape, while excluding possible biases due to mtDNA-based studies mainly focused on single lineages or on only partial segments of the mitochondrial genome. To investigate the distribution of genetic variability within Europe and the Mediterranean Basin, a Spatial Principal Component Analysis (sPCA) was performed on Thracian and modern mtDNA sequences, by using the R software package adegenet^58^. Contrary to classic PCA where eigenvalues are calculated by maximizing variance of the data, in sPCA analysis the eigenvalues are obtained by maximizing the product of variance and spatial (Moran’s I index) autocorrelation^58^. To test the significance of the detected sPCA geographical structures the Global and Local random tests implemented in the adegenet functions have been applied.

In order to diachronically compare the genetic data of Thracians with ancient population patterns, whole mitochondrial genomes of 417 ancient individuals belonging to European and Mediterranean population groups, ranging from the Upper Paleolithic to the Iron Age, were accessed through publicly available datasets (Supplementary Table S2)^18,30^. The available ancient mitogenomes were classified into geographically and culturally distinct population groups, as detailed in Supplementary Table S2. Phylogenetic relationships between ancient sequences were assessed through a Median Joining Network analysis. Sequence alignment was performed with the DNA Alignment software (www.fluxus-engineering.com) and checked manually. The Median Joining Network was calculated with the Network software v.5 (www.fluxus-engineering.com) setting the ε value to 0 and weighting the transversions 3x the weight of the transitions. The resulting network was drawn without pre- or post-processing steps and graphically visualized with Network Publisher. To summarize the relationships of Thracians with the other ancient populations, a correspondence analysis (COA) was performed by using the dudi.coa function of the R software package ade4^59^. Ancient population groups with N<5 were excluded from the analyses in order to avoid possible biases due to low population sizes.

## Supplementary information

Dataset 1

Dataset 2

## Data Availability

Mitochondrial DNA genome sequences reported in this study were submitted to NCBI GenBank (https://www.ncbi.nlm.nih.gov/genbank/) under the Accession Number MH605025-MH605049.
